# Lab-Based Evaluation of Device-Free Passive Localization Using Multipath Channel Information †

**DOI:** 10.3390/s21072383

**Published:** 2021-03-30

**Authors:** Jonas Ninnemann, Paul Schwarzbach, Andrea Jung, Oliver Michler

**Affiliations:** Institute of Traffic Telematics, Dresden University of Technology, 01069 Dresden, Germany; paul.schwarzbach@tu-dresden.de (P.S.); andrea.jung@tu-dresden.de (A.J.); oliver.michler@tu-dresden.de (O.M.)

**Keywords:** Device-Free Passive Localization (DFPL), Channel Impulse Response Environmental Mapping (CIR-EM), Wireless Sensor Networks (WSN), Ultra-Wide Band (UWB)

## Abstract

The interconnection of devices, driven by the Internet of Things (IoT), enables a broad variety of smart applications and location-based services. The latter is often realized via transponder based approaches, which actively determine device positions within Wireless Sensor Networks (WSN). In addition, interpreting wireless signal measurements also enables the utilization of radar-like passive localization of objects, further enhancing the capabilities of WSN ranging from environmental mapping to multipath detection. For these approaches, the target objects are not required to hold any device nor to actively participate in the localization process. Instead, the signal delays caused by reflections at objects within the propagation environment are used to localize the object. In this work, we used Ultra-Wide Band (UWB) sensors to measure Channel Impulse Responses (CIRs) within a WSN. Determining an object position based on the CIR can be achieved by formulating an elliptical model. Based on this relation, we propose a CIR environmental mapping (CIR-EM) method, which represents a heatmap generation of the propagation environment based on the CIRs taken from radio communication signals. Along with providing imaging capabilities, this method also allows a more robust localization when compared to state-of-the-art methods. This paper provides a proof-of-concept of passive localization solely based on evaluating radio communication signals by conducting measurement campaigns in an anechoic chamber as a best-case environment. Furthermore, shortcomings due to physical layer limitations when using non-dedicated hardware and signals are investigated. Overall, this work lays a foundation for related research and further evaluation in more application-oriented scenarios.

## 1. Introduction

Wireless Sensor Networks (WSN) are widely used and implemented in various use cases, like Internet of Things (IoT) or Indoor Positioning, and therefore open a variety of different application fields, especially in traffic telematics. In general, the employed sensors and associated communication devices measure different physical conditions of the environment and, with the purpose of further processing, collect this information at a central point. Communication between devices is realized using different technologies like Bluetooth, Wi-Fi, Ultra-Wide Band (UWB) or prospectively 5G/6G.

Next to providing an interconnection of devices, communication signals can also be used for obtaining locally available spatial information by interpreting radio signal properties. Obtaining this information of devices enables so-called location-based services (LBS) [1]. Often, these radio technologies are deployed in indoor environments, leading to the terminology Indoor Positioning Systems (IPS) [2,3]. Typically, these systems use different signal properties, like the Received Signal Strength Indicator (RSSI), Phase of Arrival (POA), Angle of Arrival (AOA) or Time of Flight (TOF) measurements to estimate the position of a mobile node in a network of stationary anchors.

For many applications, the person or object, which is supposed to be localized, is typically equipped with a transponder, leading to the term active or cooperative localization. In addition, radar-like approaches, also referred to as device-free passive localization (DFPL) [4,5,6], can use the Channel Impulse Response (CIR) of the communication channel to identify signal reflections and the associated path lengths, enabling a passive localization of reflecting objects. For the work presented, we emphasize the utilization of commonly available radio technologies originally intended for the interconnection of devices. However, the accuracy and resolution of DFPL approaches are highly dependent on physical constraints of the communication technology, for example, available bandwidth. Hence, we propose the usage of UWB radio devices, as UWB is already a key-enabler for accurate LBS, providing a widespread market penetration.

**Figure 1:**
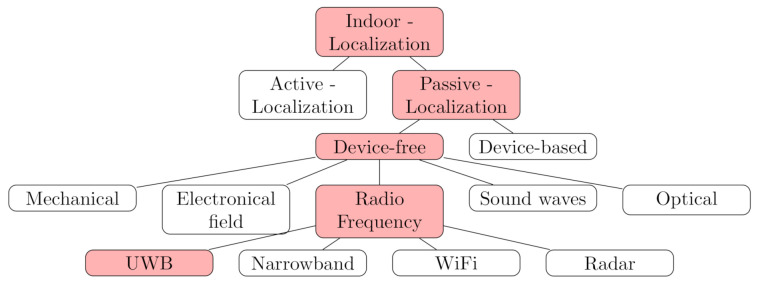
Classification of Indoor Positioning techniques, emphasizing Ultra-Wide Band (UWB)-based device-free passive localization (DFPL) [8].

Figure 1 gives a classification of common IPS approaches with the focus on radio-based DFPL approaches as well as linked radio technologies. A more detailed overview of different algorithms and techniques are provided in reference [7].

### 1.1. Status Quo of Radio Based Device-Free Localization

DFPL using only radio communication systems is an emerging research field. References [4,7,9,10] provide an overview on recent advances and challenges for radio-based DFPL. Most recently the passive object detection and sensing is also discussed for 6G [11] and the used mmWave [12].

A common approach for solving DFPL problem formulations is the utilization of an elliptical model [13,14,15,16,17]. As an example, this approach was used for radar imaging within the *Witrack* system [18,19]. The system uses dedicated and software defined radio generated frequency-modulated continuous-wave signals at a frequency around 5–6 GHz. The presented system enables through the wall object detection and breathing sensing. In contrast to this, our work aims at providing environmental perception using consumer level technologies and hardware.

Regarding technological comparisons, only UWB impulse radars have been considered in the field of passive localization so far [20]. Furthermore, related works especially address the localization and filtering of moving targets [21,22]. Next to UWB, narrow-band communication technologies have previously been used for DFPL, providing an even higher market penetration. In Ninnemann et al. [23], we conducted a DFPL approach based on IEEE 802.15.4 modules. The used 2.4 GHz ISM band is the basis of technologies like Wi-Fi, Bluetooth or Zigbee and is widely used for many applications. In the aforementioned work, the CIR is provided through an Inverse Fast Fourier Transformation (IFFT), obtained from the measured signal raw data in frequency domain. A major drawback of narrow-band technologies are the distinctiveness of different reflection paths. This is due to bandwidth constraints, leading to a low time domain resolution. Hence, UWB with broad spectrum spreading is the technology of choice in this work, since physical constraints of the communication technology drastically limit the time resolution of the CIR. However, the proposed method could potentially be implemented in a variety of already existing IoT applications.

Furthermore, several research works in the field of radio-based environmental mapping is based on RSSI measurements, which can be used for Fingerprinting or Radio Tomographic Imaging (RTI) [24,25,26]. These approaches require a comparably high device coverage or an extensive precomputation (e.g., a learning phase).

### 1.2. Focus and Structure of the Document

In our approach, a minimal setup of communication devices can be used. Additionally, we use both signal strengths and time delays of received impulses, including reflections, which are stored in the CIR. For active localization IPS, these reflections, commonly referred to as multipath propagation, are a major cause of inaccuracies. Therefore, the proposed method can also be beneficial for active localization tasks, as sources of reflections can potentially be identified.

Summarizing, we identify two major contributions in this paper—we discuss properties of radio communication technologies regarding their remote sensing capabilities and specifically present integration steps and physical constraints given the UWB technology, which is already commonly applied for active localization. In addition, we propose an environmental mapping approach, which we will refer to as Channel Impulse Response environmental mapping (CIR-EM), based on measured signal reflections. For this, we use the CIR of commercially available UWB development kits and passively identify potential reflection locations within the propagation environment, utilizing both the time delays and the received amplitudes stored within the CIR. Providing a proof-of-concept study for the proposed setup, including an experimental and quantitative CIR-EM validation as well as empirically discussing possible technological limitations is achieved by conducting a measurement campaign in an anechoic chamber.

The remainder of the article is structured as follows—after the introduction (Section 1) we discuss necessary fundamentals for signal processing and passive localization in Section 2, which are required for the realization of the CIR-EM method. Subsequently, Section 3 presents the employed hardware and all data processing steps. In Section 4 the quantitative results of the proposed DFPL system are presented and discussed. The paper concludes with a summary and proposals for future research work in Section 5.

## 2. Fundamentals

### 2.1. Channel Impulse Response

The propagation of radio signals is prone to a variety of propagation phenomena, including signal reflection, diffraction and scattering. In general, the output x(t) of a wireless communication system in form of the CIR h(t) with respect to the input signal s(t) can be described via Equation (1). The Gaussian white noise v(t) of the signal is characterized with a normal distribution $v\left(t\right)∼N\left(0,\sigma^{2}\right)$ [27].

(1) x(t)=h(t)∗s(t)+v(t)=∫h(τ)s(t−τ)dτ+v(t).

Given time-discrete impulse signals as input, the CIR represents the behavior of the communication channel in time domain and mathematically describes the superposition of various signal paths caused by reflections within the communication environment. The CIR is a complex number representing the in-phase and quadrature components of the radio signal and consists of *k* time shifted impulses $\delta (t-\tau_{k})$ represented by the delta function δ(·). It is defined in Equation (2), where $\alpha_{k}$ and $\tau_{k}$ denote the impulse amplitude and reception time delay [27,28].

(2) $$h\left(t\right)=\sum_{k=1}^{K}\alpha_{k}\delta \left(t-\tau_{k}\right).$$

**Figure 2:**
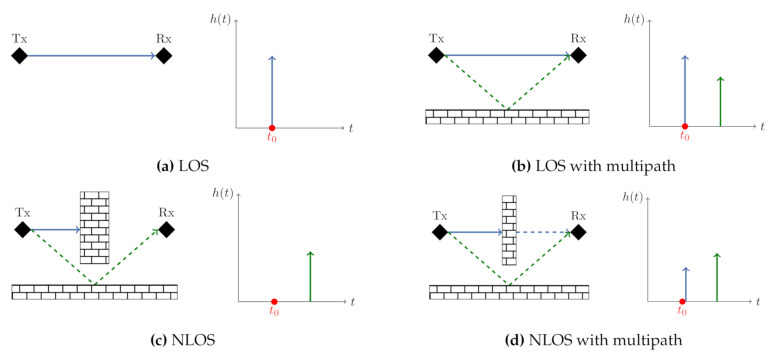
Different Propagation effects with the corresponding CIR [29]. (**a**) LOS (**b**) LOS with multipath (**c**) NLOS (**d**) NLOS with multipath.

For many real-world applications and especially in indoor scenarios, the communication channel is affected by multipath propagation, which is caused by different effects like reflections, refraction while penetrating into a medium, scattering, diffraction or absorption. The direct path between the signal transmitter and receiver is referred to as line-of-sight (LOS) propagation. In contrast, an obstructed or reflected path is called non-line-of-sight (NLOS). The term multipath reception applies, if the signal reaches the receiver via multiple paths caused by the aforementioned propagation phenomena. This is typically the case in real-life application scenarios. Figure 2 gives a general example on how the communication environment can affect the resulting CIR.

In Section 4 we will provide examples of measured CIRs within the described environment. In regard to the proposed DFPL approach, each individual path within the CIR after the direct path possibly represents a reflection of localizable object.

Obtaining the CIR can be realized by measuring the received signal and correlating it with the transmitted signal in time domain. A technology that offers various advantages for both the active and the passive radio localization approaches is Ultra-Wide Band (UWB), which is characterized by utilizing bandwidths larger than 500 MHz with frequencies ranging from 3.5 GHz to 9.5 GHz. Due to these comparably large bandwidths and the limited power spectral density, UWB is more resistant against fading related to multipath environments and jamming when compared to narrow-band technologies [30]. Common applications are short range communication, ranging between multiple sensors and indoor localization [31,32].

The impulse radio implemented in the UWB Standard IEEE 802.15.4a [31] enables a radar-like detection of the environment by providing the CIR. For this, a sequence of pulses with the pulse duration $\tau_{p}$ is used. Due to a reciprocal relation between $\tau_{p}$ and the bandwidth $B_{s}$ of the signal, a comparably short pulse duration is provided [33].

In the time domain, the range resolution *R* defines the ability to distinguish between targets and depends on the used bandwidth $B_{s}$ of the signal following Equation (3). Given an UWB channel ($B_{s}=499.2\;M\mathrm{Hz}$) the range resolution is 0.3 m or in time domain 1.0016 ns [27,34,35].

(3) $$R=\frac{c}{2\times B_{s}}.$$

Caused by the signal travel time and attenuation, the transmitted pulses are received time-delayed and with a lower amplitude at the receiver. Both of this information will further on be used for the proposed CIR-EM method. With the help of the TOF Δt it is possible to estimate the distance *d* between the transmitter and receiver via the speed of light *c*: d=Δt×c. This coherence is also used to transform the time-delay index of the CIR into the distance index [33].

### 2.2. Passive Localization

#### 2.2.1. Problem Statement

As an input for the proposed passive localization method, every value in the CIR except the direct path is used, as each following peak represents a potential reflection at a target object. This allows the determination of the lengths of the indirect paths $d_{i}$, which are the sum of the distances between the transmitter and target object $r_{\mathrm{Rx}}$ as well as the distance between the target object and the receiver $r_{\mathrm{Tx}}$ with a measurement error $\varepsilon_{i}$:

(4) $$d_{i}=r_{\mathrm{Rx}}+r_{\mathrm{Tx}}+\varepsilon_{i}=\Delta t\times c.$$

**Figure 3:**
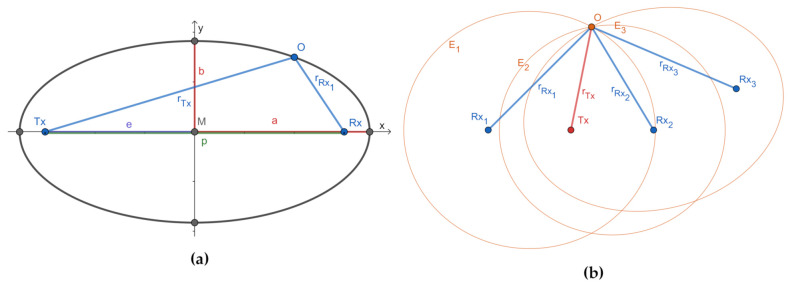
Ellipses visualization: (**a**) parameters of an ellipse; (**b**) ellipses intersection within a Wireless Sensor Networks (WSN).

The positions of the transmitter $X_{\mathrm{Tx}}\;=\;{\left[x_{\mathrm{Tx}},y_{\mathrm{Tx}}\right]}^{⊺}$ and *n*-receivers $X_{\mathrm{Rx},i}\;=\;{\left[x_{\mathrm{Rx},i},y_{\mathrm{Rx},i}\right]}^{⊺}$ (i=1,⋯,n) within the communication network have to be known in order to estimate the position of the target object ${\hat{X}}_{O}=\left({\hat{x}}_{O},{\hat{y}}_{O}\right)$. Based on Equation (4), a non-linear equation system as given in Equation (5) can be formulated. This equation system describes the lengths of the reflection paths caused by an object within the coverage of the applied sensor network (cf. Figure 3) [13,14].

(5) $$d_{i}=\sqrt{{\left(x_{\mathrm{Tx}}-{\hat{x}}_{O}\right)}^{2}+{\left(y_{\mathrm{Tx}}-{\hat{y}}_{O}\right)}^{2}}+\sqrt{{\left(x_{\mathrm{Rx},i}-{\hat{x}}_{O}\right)}^{2}+{\left(y_{\mathrm{Rx},i}-{\hat{y}}_{O}\right)}^{2}}+\varepsilon_{i}\;i=1,\cdots ,n.$$

Since WSN typically cover a limited spatial area, the transmitter and receiver are separated by a distance similar to the distances to the objects to be located. Therefore, the proposed setup corresponds to a bistatic radar system. The constant transmitter to target to receiver range *d* obtained by the bistatic radar is equal to double of the semi-major axis of an ellipse *a* as given in Equation (6) [16]. The transmitter and the receiver are situated in the foci of the ellipse and the target object is located somewhere on the ellipse, as the ellipse describes all points with a constant sum of the reflection path between transmitter, receiver and possible target object locations. In addition to the semi-major axis of the ellipse, the semi-minor axis *b* is also obtained from the direct LOS path length *p* between transmitter and receiver.

(6) $$d=2a\;\;\;\;b=\sqrt{a^{2}-{\left(\frac{p}{2}\right)}^{2}}.$$

The semi-major axis and semi-minor axis allow the formulation of the ellipse equation in parametric form. Thereby for every measured reflection path length and every transmitter to receiver relation an ellipse with the corresponding parameters can be established. For the position estimation this problem statement can be solved by explicitly calculating elliptical intersections (Figure 3b) [14,17].

An alternative solution approach for the non-linear equation given in Equation (5) is the least squares method, for example, the Gauss-Newton method. Here the function is linearized at a working point using Taylor’s theorem. The solution of the resulting linear least-squares problem is used to adjust the position estimation in an iterative process [13,14]. Both methods for solving the passive localization problem will be used in Section 4 as a comparison regarding the positioning performance.

#### 2.2.2. Channel Impulse Response Environmental Mapping

**Figure 4:**
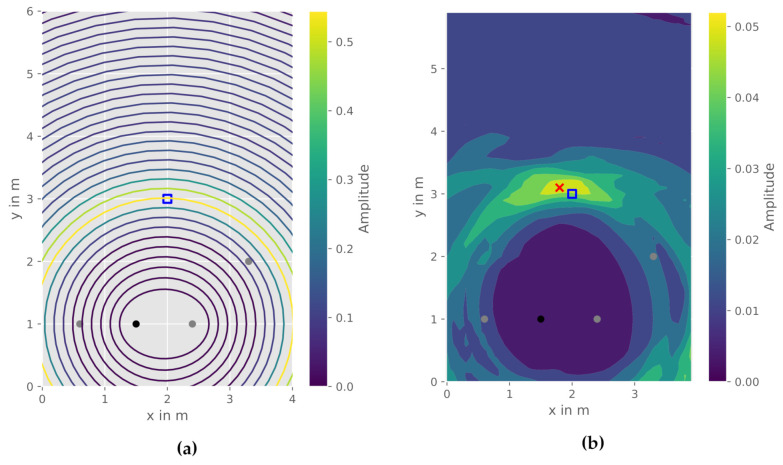
CIR-EM, reference object position (blue square), estimated object position (red), transmitter (black), receiver (grey): (**a**) Family of ellipses obtained from one CIR; (**b**) Interpolated heatmap.

In contrast to the previously mentioned solution approaches to DFPL, the CIR-EM method does not only use one reflection path at possible target objects, but rather employs all values given in the CIR, as each value in the CIR is correlated to a specific time delay and length of a reflection path in the environment. Thus, to every amplitude value an ellipse *E* with a specific semi-major axis *a* can be calculated. The result is a family of ellipses between transmitter and receiver (Figure 4a). With the help of the ellipses, the CIR amplitude data is plotted in a two-dimensional plane. An interpolation over an equidistant grid creates a continuous value surface. This grid is calculated for every measured CIR in the WSN. The final heatmap is generated by averaging the grids for all CIR measurements between all communication nodes. Both the obtained family of ellipses and the resulting heatmap interpolation are depicted in Figure 4b. The object position ${\hat{X}}_{O}$ can be estimated from the heatmap by finding the grid cell with the highest amplitude. In general, a higher amplitude in the heatmap represents quantitatively more respectively stronger reflections from an object. Potentially, the size of the object is also correlated to the area with higher amplitudes, if the time resolution of the CIR is high enough. Algorithm 1 provides an overview of all calculation steps for the CIR-EM method. The main computation steps consist of two loops which iterate over all CIR between every transmitter $X_{\mathrm{Tx}}$ and receiver $X_{\mathrm{Rx}}$ and over all data points within the corresponding amplitude $\alpha_{k}$ in every CIR. The processing steps of the CIR-EM is comparable to radar imaging, where the communication module respectively the radar is used to create a two-dimensional image of the environment [36].
**Algorithm 1:** Channel Impulse Response Environmental Mapping (CIR-EM).
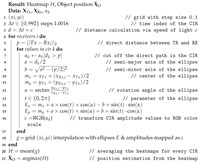


## 3. Integration and Processing

**Figure 5:**
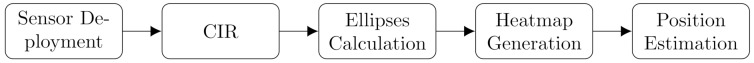
Flowchart of data processing and computation steps for CIR-EM.

This section introduces the used hardware and processing steps required to implement and validate the CIR-EM method. Figure 5 depicts a flowchart of all computation steps.

The applied sensor setup and the corresponding measured CIRs will further be discussed in the following subsections. The ellipses calculation, heatmap generation and position estimation refer to the CIR-EM approach discussed in Section 2.

### 3.1. Sensor Deployment-DecaWave EVB 1000

The EVB1000 sensors board from *DecaWave (Dublin, Ireland)* is a part of the TREK1000 Evaluation Kit and is equipped with the STM32 microcontroller and the DW1000 UWB chip [37]. This chip implements the UWB Standard and therefore allows different applications of the sensor, for example, Two-Way Ranging, Real-time locating systems or tracking. The microcontroller communicates via SPI with the DW1000 and is programmable with the firmware.

For the use case of passive object detection, the CIR is directly measured by the UWB communication modules and forwarded to the PC via USB. This is achieved by modifying the firmware of the modules using the provided *DecaWave* API [37], which enables a star topology for the network. The CIR is saved in the accumulator memory along with some diagnostic data in other registers of the chip. According to [37], the DW1000 provides a two-dimensional localization accuracy of ±30 cm. The measurements conducted in this work were recorded using the UWB channel 4, providing a channel bandwidth $B_{S}$ of 1331.2 MHz and a data rate of 850 kbps.

In addition, the Pulse Repetition Frequency (PRF) $T_{p}$ is a principal parameter of the impulse radio. The PRF is the reciprocal of the Pulse Repetition Interval (PRI) $f_{p}=1/T_{p}$. The PRI describes the time between two transmitted pulses. The PRF determines the range resolution of the radio. Every single pulse has to be separately identifiable before the next impulse can be sent [33]. The UWB standard specifies the PRF with 16 MHz or 64 MHz. Due to the PRF, the duration of one symbol is 993 ns or 1018 ns, which results in the CIR consisting of 992 respectively 1016 evenly spaced values [31]. For the measurement setup a PRF of 16 MHz with a preamble length of 1024 and a PAC size of 32 was applied.

### 3.2. CIR Raw Data

The *DecaWave* EVB1000 UWB modules forwards the complex CIR raw data via the serial port to the host PC. The length of the data array depends on the applied PRF. Each value of the CIR is separated by 1 ns regarding the available bandwidth of the signal (Equation (3)) [35]. The complex raw data is used to calculate the amplitude of the CIR. To estimate the length of the reflection paths from the CIR, all values before the direct path between the transmitter and the receiver are removed in the CIR. The remaining values are then further processed as described in Section 2.

## 4. Results & Discussion

**Figure 6:**
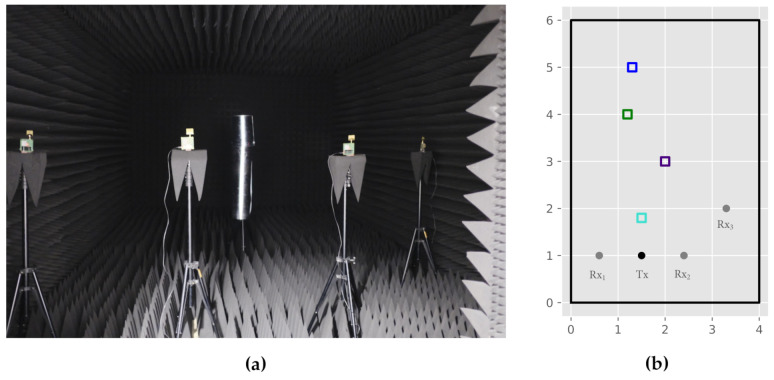
Measurement setup: (**a**) Anechoic chamber; (**b**) Sensor arrangement: receivers (grey), transmitter (black) and object positions: $O_{1}$ (blue), $O_{2}$ (green), $O_{3}$ (purple) and $O_{4}$ (lightblue).

A variety of influencing factors in terms of positioning accuracy for DFPL and the proposed CIR-EM exist. Since the underlying elliptical problem formulation used signal reflections as its main input, the recognizability of reflections within the CIR is a key factor for the subsequent positioning process. The CIR recognizability in turn mainly depends on the following aspects: shape and material characteristics of the reflection object, hardware range resolution combined with the lengths of the reflection paths as well as geometric network constellation. Within the contributed measurement campaign, we want to address and focus the latter two influences. To still ensure reasonable reflection properties of the reflecting object, a metallic and cylindrical object with a size of 24 cm × 30 cm is investigated (Figure 6). This shape also provides the best properties in terms of reflection, as a close to uniform reflection is present.

The scenarios examined in this paper serve to evaluate the following objects of investigation:

- Sensor distribution and object position, as well as its recognizably within the CIR and
- Accuracy assessment for the proposed CIR-EM method for one constellation.

The latter is achieved by determining the two-dimensional offset between the true $X_{\mathrm{true}}$ and the estimated ${\hat{X}}_{O}$ target object position. The measures used are the root mean square error (RMSE) and the mean absolute error (MAE), which for k=1,⋯,N measurement epochs are defined as:

(7) $$Q_{\mathrm{MAE}}=\frac{1}{N}\sum_{k}{\left|\left|x_{{\mathrm{true}}_{k}}-{\hat{x}}_{k}\right|\right|}_{1}\;\;\;\;Q_{\mathrm{RMSE}}=\sqrt{\frac{1}{N}\sum_{k}{\left|\left|x_{\mathrm{true}}-{\hat{x}}_{k}\right|\right|}_{2}}$$

In order to provide a reconstructable experimental setup, the measurements were conducted in a radio-frequency anechoic chamber. This environment offers two major benefits for validation—reflections within the room, for example, by walls or other objects are minimized. Additionally, there are no radio interferences caused by other communication devices. Figure 6a depicts the anechoic chamber, the employed communication modules as well as the target object.

### 4.1. WSN Constellation

**Figure 7:**
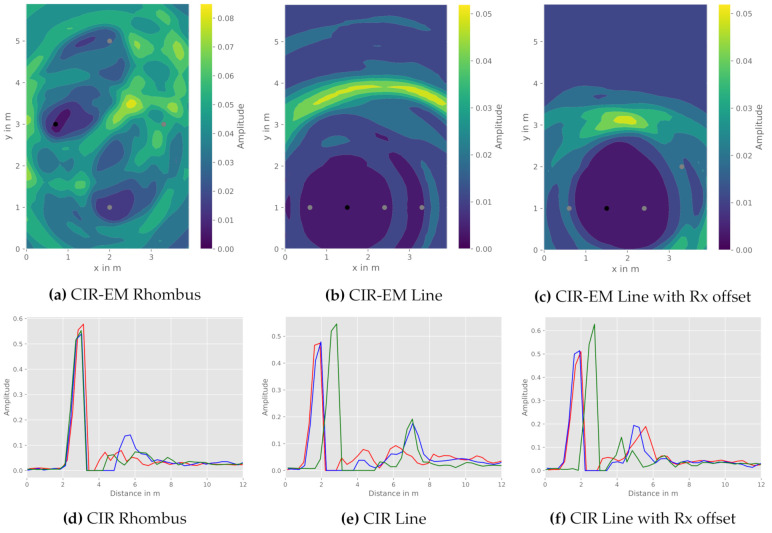
Comparison of the CIR-EM with different sensor constellations: transmitter (black), receiver (grey), CIR Tx-${\mathrm{Rx}}_{1}$ (red), CIR Tx-${\mathrm{Rx}}_{2}$ (blue), and CIR Tx-${\mathrm{Rx}}_{3}$ (green). (**a**) CIR-EM Rhombus (**b**) CIR-EM Line (**c**) CIR-EM Line with Rx offset (**d**) CIR Rhombus (**e**) CIR Line (**f**) CIR Line with Rx offset.

Since the work presented relies on communication modules, physical constraints in terms of bandwidth and accompanying range resolution are responsible for the amount of discrete CIR values, possibly hindering reflection detectability. This effect can specifically be observed, if the length of the direct and the reflected path are similar in amount. In order to assess this effect, Figure 7 depicts three exemplary transmitter to receiver constellations and their corresponding CIR.

As highlighted in the visualization of the CIR-EM results, several issues in terms of reflection recognizability for the different constellations arise. At first, Figure 7a,d depict a rhombus-shaped constellation with a centered object position. Due to the distance likeliness of the direct and the reflected path, the ambiguity effect of not being able to differ between reflection paths, as a result of limited range resolution, becomes apparent. This leads to insignificant CIR-EM results. While the CIR-EM approach is utilized for visualizing this effect, the other aforementioned methods, namely the Gauss-Newton and the intersection method, suffer from the same shortcomings.

In order to increase the ability to distinguish between different signal paths in the CIR and due to spatial restrictions of the anechoic chamber, a linear WSN constellation was also examined. This allows a further separation of signal path lengths (Figure 7e), a linear constellation presents a comparably high geometric dilution of precision (GDOP), which can be seen in Figure 7b, and thus leads to inaccuracies in the positioning process, as the spatial resolution is constrained. Hence, further accumulation points due to ambiguities can occur.

In order to overcome the disadvantages of aforementioned constellations, a third constellation was examined. A slight GDOP improvement, while meeting range resolution constraints, can be achieved by rearranging one receiver apart from the linear constellation as seen in Figure 7c,e. During the surveyed experiments, this constellation provided the best recognizability of reflections within the CIR and hence is further investigated for different reflection object positions.

### 4.2. Reflection Object Positions

**Figure 8:**
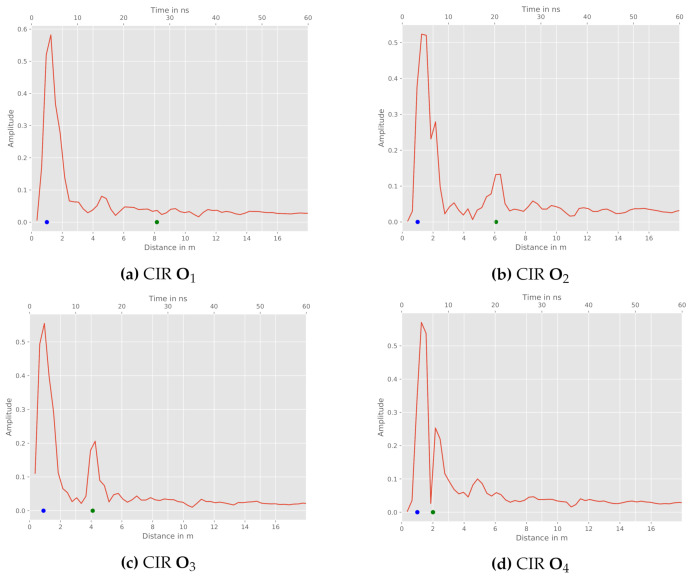
Tx and ${\mathrm{Rx}}_{2}$ CIRs for different object positions: direct path (blue), reference reflection (green). (**a**) CIR $O_{1}$ (**b**) CIR $O_{2}$ (**c**) CIR $O_{3}$ (**d**) CIR $O_{4}$.

Besides the WSN arrangement and the aforementioned influences, the position of the reflection object in relation to the sensor constellation must be examined. In order to do so, the constellation and object positions depicted in Figure 6b are further investigated. For the chosen setup, we exemplary depict the measured CIRs between Tx and ${\mathrm{Rx}}_{2}$ for all four object positions in Figure 8.

Firstly, object $O_{1}$ shows no characteristic peak within the CIR (cf. Figure 8a). Hence, this measurement does not significantly contribute to the position estimation. Possible reasons are the range resolution of the sensor and the size of the reflecting object being too small in this case. The further object positions provide a characteristic peak within the CIR and therefore a recognizable reflection. This is also indicated with green dots, which mark the reference reflection path length for the respective relation. On the downside, object position $O_{4}$ leads to a proximate reflection near the sensors. Figure 8d reveals nearly the same length of the direct path and the reflection path at the object.

Based on these inputs, the accuracy performance of the proposed CIR-EM method is compared to a conventional geometric elliptical intersection calculation and an iterative Gauss-Newton approach. All obtained quantitative positioning accuracy results are presented in Table 1, including the average errors and variances for the RMSE and MAE. In addition, the 68.27%, 95.45% and 99.73% quantiles of the empirical cumulative density functions (ECDF) as well as the 25th, 50th and 75th percentile of all error measures are provided. The measurements of the CIR raw data were performed in a static environment over 1000 iterations for each constellation.

**Figure 9:**
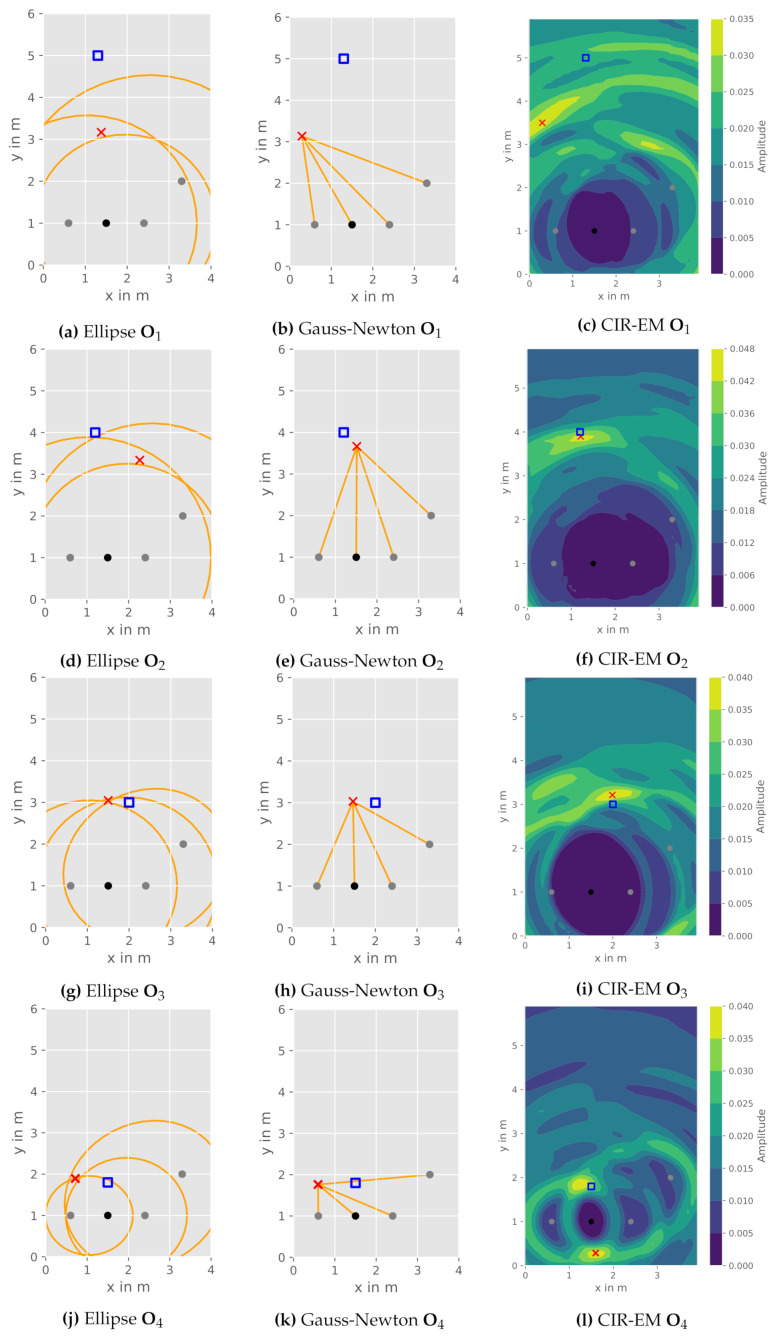
Comparison of DFPL approaches for different object positions: object reference position (blue square), estimated object position (red cross), transmitter (black), receivers (grey).

In addition to the quantitative evaluation, we depict and discuss the influences of the reflection object position given the presented constellation (Figure 9) for both the CIR-EM as well as the aforementioned elliptical intersection and Gauss-Newton method.

The geometrical elliptical intersection works well if all ellipses intersect nearly in one point. An example is observable for the object position $O_{3}$ in Figure 9g. Though, this approach fails with intersection points at different locations caused by a flawed measurement of the reflection path. A decision between the points is not possible and averaging is not expedient due to the occurring positional ambiguities caused by measurement errors (Figure 9d).

Subsequently, the Gauss-Newton method does not struggle with multiple intersection points, as it estimates the object position with help of a numeric approach. This leads to a slightly better performance of the object position $O_{2}$ in Figure 9e. Nevertheless, the elliptical intersection and the Gauss-Newton algorithm perform similarly, as they essentially solve the same equation system utilizing two different deterministic (geometric and numeric) approaches.

The CIR-EM approach results in a better performance for the measurements at object position $O_{2}$ and $O_{3}$ (Figure 9f,i), due to the consideration of all values within the CIRs. A potential shortcoming of this method can be depicted in Figure 9l, where the reflection is mapped to two areas in the heatmap. This is caused by the aforementioned possible ambiguities for the chosen constellation. All ellipses correlating to the reflection path have nearly the same two intersection points. In this case, the averaged heatmap does not allow an accurate position estimation. In addition to generally more accurate localization results when compared to the elliptical intersection and the Gauss-Newton method, the CIR-EM approach also allows radar imaging of the environment. Therefore, it enables the mapping of reflections from multiple objects in the room.

**Figure 10:**
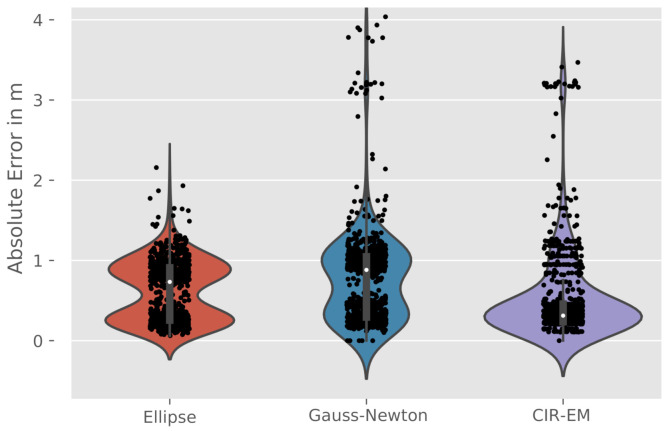
Violinplots of the absolute error for the different algorithms at $O_{3}$.

Furthermore, the distribution of the absolute error for the object position at $O_{3}$ is depicted in Figure 10. Here the elliptical intersection and the Gauss-Newton method results reveal two peaks in the error distribution, whereas the CIR-EM method follows an unimodal distribution. This backs up the formulated assumptions on the lack of robustness of the elliptical intersection and the Gauss-Newton method given the presented scenario. The CIR-EM, as a result of the interpolation over the grid and consideration of all CIR values, is a more robust approach in the validated scenarios when compared to the aforementioned ones. However, it is assumed, that the CIR-EM method is connected to higher computational complexities.

Concluding, the accuracy of all mentioned localization methods mainly depends on the quality of the measurement raw data, the range resolution and geometric constellation of the sensors, the object shape and material properties (especially reflection characteristics), as well as the radio propagation environment.

## 5. Conclusions & Outlook

In this contribution, a radar-like DFPL object detection and localization, solely based on radio communication signals, is addressed. Due to the applicability of different communication standards and protocols, the discussed DFPL approaches yield a broad variety of practical use cases, especially in the context of traffic telematic environments and IoT applications. Depending on the targeted usage and its demands in terms of localization accuracy, equipment level and range, different radio technologies and protocols can be used. A widespread technology for high precision applications is UWB, as it offers a comparably robust ranging performance even in challenging environments.

In addition to active localization approaches, which require the users or objects to carry dedicated devices, our contribution was focused on providing location information solely based on signal reflections. These are measurable and represented in the CIR, which characterizes the radio communication channel between transmitter and receiver for impulse input signals. Based on these reflections, we proposed and discussed a novel positioning method, which we referred to CIR-EM, aiming to solve multipath assisted DFPL.

In order to provide a general proof-of-concept, commercially available UWB modules were used to collect CIR data in various lab-based static measurement scenarios. The measurement environment of choice was an anechoic chamber, aiming to provide reconstructable and resolvable object reflections.

Under the prevailing conditions (physical constraints of the sensors, close to ideal conditions within the anechoic chamber and only one localizable object), the measurement campaign revealed both capabilities and challenges of the proposed approach. Influential parameters, such as radio constraints (e.g., bandwidth), geometric sensor to object constellation, object size and object reflection properties influence the localization accuracy. With the focus in this work being the investigation of different transmitter to receiver to reflection object constellations, the geometric effects in combination with the given radio range resolution have been discussed. Without the usage of specialized or dedicated radar sensors, we were able to provide the functionalities of passive localization solely based on the transmission channel properties.

For future work, we plan to incorporate more robust state estimation and filtering methods to deal with multi-modal positioning distributions (Figure 10). This specifically includes probabilistic solution approaches to the described problem formulation. Also, a floor plan combined with a ray tracing simulation can be used to support and compare the CIR measurements and the corresponding multipath.

In addition, further physical layers and communication technologies will be examined. This includes additional wireless technologies, like 5G/6G or light fidelity (Li-Fi). Also, the impact of different antenna systems and their optimization for the DFPL should be evaluated. The usage of a beam forming antenna could potentially improve the robustness and accuracy of the localization by eliminating unwanted multipath.

In concern to realistic applicability, we will conduct and evaluate CIR measurements in different environments and possible application fields. For these types of scenarios, we identify two necessary steps: separation of static and dynamic reflections via a filter and background subtraction in the CIR. Essentially, the next important step is to leave the close to ideal environment of the anechoic chamber and to evaluate our proposed DFPL setup in realistic scenarios.

## Figures and Tables

**Table 1:** Accuracy assessment, including RMSE and MAE as well as associated variances, empirical cumulative density functions (ECDF) quantiles and percentiles, of the proposed passive localization methods for different constellations.

Position	Method	RMSE								MAE							
		Q	$\sigma^{2}$	Quantile[m]			Percentile[m]			Q	$\sigma^{2}$	Quantile[m]			Percentile[m]		
		[m]	${\left[m\right]}^{2}$	68.27%	95.45%	99.73%	**25**	**50**	**75**	[m]	${\left[m\right]}^{2}$	68.27%	95.45%	99.73%	**25**	**50**	**75**
$O_{1}$	Ellipse	2.02	0.05	2.16	2.35	2.57	1.80	2.03	2.19	2.25	0.14	2.49	2.73	2.97	1.81	2.31	2.54
	Gauss-Newton	3.61	4.77	6.40	6.49	6.50	1.80	2.12	6.43	3.76	4.44	6.44	6.51	6.53	1.97	2.45	6.47
	CIR-EM	2.02	0.50	2.14	2.32	4.91	1.98	1.76	2.74	2.31	0.50	2.50	2.75	5.06	1.93	2.06	2.57
$O_{2}$	Ellipse	1.06	0.19	1.32	1.49	1.52	1.09	1.21	1.34	1.23	0.26	1.52	1.75	1.78	1.26	1.40	1.55
	Gauss-Newton	0.53	0.50	0.43	0.74	4.72	0.38	0.42	0.46	0.60	0.56	0.51	0.83	5.03	0.43	0.48	0.54
	CIR-EM	0.21	0.06	0.24	0.32	0.94	0.10	0.19	0.25	0.24	0.08	0.29	0.37	1.02	0.11	0.19	0.29
$O_{3}$	Ellipse	0.59	0.13	0.84	1.11	1.45	0.22	0.69	0.88	0.62	0.14	0.87	1.15	1.50	0.26	0.73	0.91
	Gauss-Newton	0.75	0.33	0.98	1.42	3.21	0.27	0.85	1.02	0.78	0.35	1.01	1.50	3.26	0.30	0.89	1.05
	CIR-EM	0.48	0.26	0.36	1.35	2.93	0.22	0.30	0.42	0.52	0.30	0.42	1.39	3.20	0.24	0.31	0.46
$O_{4}$	Ellipse	0.51	0.18	0.47	1.23	1.24	0.19	0.37	0.92	0.54	0.20	0.51	1.29	1.30	0.19	0.38	0.94
	Gauss-Newton	0.75	0.27	0.72	1.63	2.05	0.35	0.62	0.86	0.80	0.30	0.80	1.85	2.22	0.35	0.71	0.89
	CIR-EM	1.01	0.42	1.51	1.62	1.82	0.29	1.45	1.53	1.07	0.48	1.59	1.86	2.01	0.30	1.50	1.61

## Data Availability

Not applicable.
